# Impact of frailty on short term outcomes, resource use, and readmissions after transcatheter mitral valve repair: A national analysis

**DOI:** 10.1371/journal.pone.0259863

**Published:** 2021-11-18

**Authors:** Joseph Hadaya, Zachary Tran, Yas Sanaiha, Esteban Aguayo, Vishal Dobaria, Marcella Calfon Press, Peyman Benharash

**Affiliations:** 1 Department of Surgery, Division of Cardiac Surgery, David Geffen School of Medicine at UCLA, Los Angeles, California, United States of America; 2 Department of Medicine, Division of Cardiology, David Geffen School of Medicine at UCLA, Los Angeles, California, United States of America; Bern University Hospital, SWITZERLAND

## Abstract

**Background:**

Treatment options for mitral regurgitation range from diuretic therapy, to surgical and interventional strategies including TMVR in high-risk surgical candidates. Frailty has been associated with inferior outcomes following hospitalizations for heart failure and in open cardiac surgery.

**Objective:**

The purpose of the present study was to evaluate the impact of frailty on clinical outcomes and resource use following transcatheter mitral valve repair (TMVR).

**Methods:**

Adults undergoing TMVR were identified using the 2016–2018 Nationwide Readmissions Database, and divided into *Frail* and *Non-Frail* groups. Frailty was defined using a derivative of the Johns Hopkins Adjusted Clinical Groups frailty indicator. Generalized linear models were used to assess the association of frailty with in-hospital mortality, complications, nonhome discharge, hospitalization costs, length of stay, and non-elective readmission at 90 days. Average marginal effects were used to quantify the impact of frailty on predicted mortality.

**Results:**

Of 18,791 patients undergoing TMVR, 11.6% were considered frail. The observed mortality rate for the overall cohort was 2.2%. After adjustment, frailty was associated with increased odds of in-hospital mortality (AOR 1.8, 95% CI 1.2–2.6), corresponding to an absolute increase in risk of mortality of 1.1%. Frailty was associated with a 2.7-day (95% CI 2.1–3.2) increase in postoperative LOS, and $18,300 (95% CI 14,400–22,200) increment in hospitalization costs. Frail patients had greater odds (4.4, 95% CI 3.6–5.4) of nonhome discharge but similar odds of non-elective 90-day readmission.

**Conclusions:**

Frailty is independently associated with inferior short-term clinical outcomes and greater resource use following TMVR. Inclusion of frailty into existing risk models may better inform choice of therapy and shared decision-making.

## Introduction

Mitral regurgitation (MR) is the second most common valvular disease in modern countries and is strongly associated with atrial fibrillation, congestive heart failure, and poor quality of life [1, 2]. Over three decades ago, Carpentier formalized the classification of mitral valve pathologies and proposed durable and tailored repair methods [3]. However, many patients at high surgical risk, such as those with reduced left ventricular function and pulmonary hypertension, have historically not been offered surgical therapy. Such patients often suffer from multiple heart failure episodes and require repeat hospitalizations for diuretic therapy.

Transcatheter mitral valve repair (TMVR) has recently emerged as an alternative to surgery for the treatment of severe symptomatic MR, using catheter-based methods to appose the two leaflets in regions of malcoaptation [4, 5]. Although randomized trials have not demonstrated an impact on life expectancy, TMVR has been found efficacious in reducing repeat hospitalizations [6]. Compared to surgical candidates, patients undergoing TMVR are often older and carry a significant burden of comorbidities, leading to suboptimal outcomes following this intervention [7, 8].

While several trials and registries have examined patient outcomes based on general preexisting conditions and laboratory data, few have studied the impact of frailty in this cohort. Despite the lack of a universal definition or assessment, frailty has been associated with poor surgical and procedural outcomes [9–11]. This may be attributable, in part, to factors such as age, chronic diseases, and an inability to withstand acute physiologic stress [12, 13]. Frailty has been shown to increase 1-year postoperative mortality in several operative categories, ranging from oncologic operations to open cardiac surgery [14–18]. However, frailty may have a less profound impact on outcomes following less invasive procedures such as TMVR. Kundi et al. used the Hospital Frailty Risk Score (HFRS) in Medicare patients undergoing TMVR and found an association between frailty and 1-year mortality rates [19]. However, this study was limited by a small sample size, as well as a frailty indicator that included many diagnoses that correspond to in-hospital complications, hindering the study’s interpretation and limiting its applicability in a priori determination of risk. Recent studies examining frailty in surgical patients have utilized the Johns Hopkins Adjusted Clinical Groups frailty indicator (ACG) and its derivatives, which are based on frailty-defining diagnoses with minimal overlap with complications and traditional procedural risk factors [14, 20, 21]. The present study aimed to assess the impact of frailty as determined by the ACG on in-hospital mortality, complications, and resource use in a national cohort of patients undergoing TMVR.

## Methods

### Data source and cohort definitions

We performed a cohort study of all adult patients who underwent TMVR from 2016 to 2018 using the Nationwide Readmissions Database (NRD) [22]. The NRD is an all-payer inpatient database maintained by the Agency for Healthcare Research and Quality (AHRQ) as part of the Healthcare Cost and Utilization Project. The NRD provides nationally representative estimates of >57% of all inpatient hospitalizations in the United States annually [22]. The NRD contains linkage numbers for all sample patients, allowing for readmissions within each calendar year to be tracked across hospitals.

*International Classification of Disease*, *Tenth Revision*, *Procedure Coding System* (ICD-10-PCS) codes were used to identify adult patients (ages ≥ 18 years) who underwent TMVR (02UG3JZ, 02UG4JZ, 02QG4ZZ, 02QG3ZZ) from 2016–2018. Years prior to 2016 were not studied due to low sample size and the transition from *International Classification of Disease*, *Ninth Revision* to ICD-10-PCS codes. Patients were divided into *Frail* and *Non-Frail* cohorts using *International Classification of Disease*, *Tenth Revision*, *Clinical Modification* (ICD-10-CM) codes corresponding to the Johns Hopkins Adjusted Clinical Groups (ACG) frailty-defining diagnoses. The indicator is derived from ten groupings of frailty-defining diagnoses including malnutrition, dementia, vision impairment, decubitus ulcer, urinary and fecal incontinence, weight loss, falls, difficulty walking, poverty, and barriers to healthcare access. The presence of one or more diagnosis groupings was used to categorize a patient as frail [20, 21].

### Variable definitions and outcomes

Patient and admission level characteristics were defined in accordance with the NRD Data Dictionary including age, sex, urgency of admission (elective versus urgent or emergent) and primary payer [22]. Similarly, hospital level variables available in NRD included bed size and teaching status. Comorbidities were further defined using ICD-10-CM codes and the Elixhauser Comorbidity Index, a previously validated numeric burden of 30 common conditions [23]. Complications were categorized into cardiac (cardiac arrest, myocardial infarction, cardiac tamponade, ventricular fibrillation, and ventricular tachycardia), pulmonary (pneumonia, pneumothorax, acute respiratory distress syndrome, respiratory failure), infectious (sepsis, septicemia, wound infection) and renal (acute kidney injury) as previously described [24]. Hospitalization costs were determined in accordance with methodology reported by HCUP [25]. Briefly, total hospital charges were converted to costs using hospital-specific cost-to-charge ratios published by the AHRQ and then inflation-adjusted to 2018 using the Bureau of Labor Statistics Consumer Price Index [26].

The primary outcomes of the study were in-hospital mortality, perioperative complications, non-home discharge and nonelective readmissions, defined as within 90-days of index hospitalization. Secondary outcomes included postoperative length of stay (LOS), adjusted hospitalization costs and diagnoses for rehospitalization. Diagnosis Related Groups (DRG) in combination with ICD codes were used to identify principal readmission diagnoses as previously described [27].

### Statistical analysis

All statistical analysis was performed with Stata 16.0 (StataCorp, College Station, TX) using survey-specific methods to account for clustering and stratification. Patients with missing age, sex, mortality data or discharge disposition, and costs were excluded from analysis (56, or 0.3% of final cohort). Continuous variables are reported as mean with standard deviation or median with interquartile range (IQR) if non-normally distributed. Categorical variables are reported as frequency or proportion. Patient and hospital characteristics were compared between cohorts using chi-square and adjusted Wald tests. Freedom from readmission was evaluated utilizing Kaplan-Meier survival analysis with the log-rank test used to assess the significance of frailty on readmission. Multivariable logistic and linear regression models were developed to evaluate the association of frailty with outcomes of interest. Elastic Net with retention of clinically-relevant characteristics was used for variable selection [28]. Briefly, Elastic Net utilizes a regressive least squares methodology to select explanatory variables aimed at reducing collinearity while applying penalties to decrease overfitting. Final models were evaluated by the area under the receiver operating characteristics curve and Akaike information criteria. Adjusted impact of independent variables are reported as adjusted odds ratios (AOR), beta-coefficients (β), and average marginal effects. To determine marginal effects, the Stata *margins* command was used to calculate point estimates and confidence intervals. Statistical significance was defined as α<0.05. This study was deemed exempt from full review by the Institutional Review Board at the University of California, Los Angeles. Specific consent from individual patients was not required due to the deidentified nature of the data set.

## Results

### Characteristics of frail and non-frail groups

Of an estimated 18,791 patients undergoing TMVR during the study period, 2,179 (11.6%) comprised the *Frail* group. Baseline characteristics of the *Frail* and *Non-Frail* groups are reported in Table 1. The most common frailty defining diagnoses were dementia (33.0%) and malnutrition (37.0%). Compared to *Non-Frail*, patients in the *Frail* group were older (78.9±10.5 years vs 77.3±10.8 years, P<0.001) and had a greater aggregate burden of comorbidities as defined by the Elixhauser Comorbidity Score (7.0±2.3 vs 5.6±2.0, P<0.001). Specifically, the *Frail* group had higher rates of congestive heart failure, coagulopathy, electrolyte disorders, but lower a lower incidence of peripheral vascular disease compared to the *Non-Frail* group (Table 1). *Frail* patients on average experienced a longer preoperative length of stay (5.3±9.4 days vs 1.3±4.0 days, P<0.001) and a greater proportion of non-elective admissions (51.7% vs 78.6%, P<0.001) compared to *Non-Frail*. There were no significant differences in hospital characteristics by frailty status with similar proportion treated by teaching status and bed size.

**Table 1:** Patient and hospital characteristics of patients undergoing TMVR from 2016–2018 by *frail* and *non-frail* cohorts.

	*Frail* (n = 2,179)	*Non-Frail* (n = 16,612)	*P-value*
Age (mean, SD)	78.9 (10.5)	77.3 (10.8)	<0.001
Female (%)	46.9	46.0	0.61
Days to procedure (mean, SD)	5.3 (9.4)	1.3 (4.0)	<0.001
Elective Admission (%)	51.7	78.6	<0.001
Income Quartile (%)			0.32
Fourth (Highest)	28.3	25.6	
Third	26.0	27.2	
Second	24.2	25.7	
First (Lowest)	21.6	21.5	
Primary Insurer (%)			0.087
Private	8.0	10.2	
Medicare	88.4	85.5	
Medicaid	2.3	2.6	
Other*	1.4	1.7	
Hospital Type (%)			0.12
Urban teaching	91.5	90.0	
Urban non-teaching	8.3	9.9	
Rural	0.20	0.12	
Hospital Bed Size (%)			0.081
Large	78.1	74.3	
Medium	19.6	21.5	
Small	2.4	4.1	
Elixhauser Comorbidity Index (mean, SD)	7.0 (2.3)	5.6 (2.0)	<0.001
Comorbidities (%)			
Cardiac arrhythmia	76.2	68.6	<0.001
Chronic lung disease	31.5	26.8	<0.001
Coagulopathy	17.9	8.9	<0.001
Coronary artery disease	61.0	62.3	0.42
Diabetes mellitus	27.5	26.3	0.4
End stage renal disease	5.79	4.44	0.043
Hypertension	84.6	81.6	0.02
Hypothyroidism	20.4	18.0	0.047
Liver disease	7.54	3.23	<0.001
Malignancy	3.23	2.44	0.092
Pulmonary hypertension	40.0	30.4	<0.001

*Other payer includes self-pay, no charge, or other as defined by the NRD.

### Unadjusted outcomes in frail versus non-frail patients following TMVR

**Fig 1:**
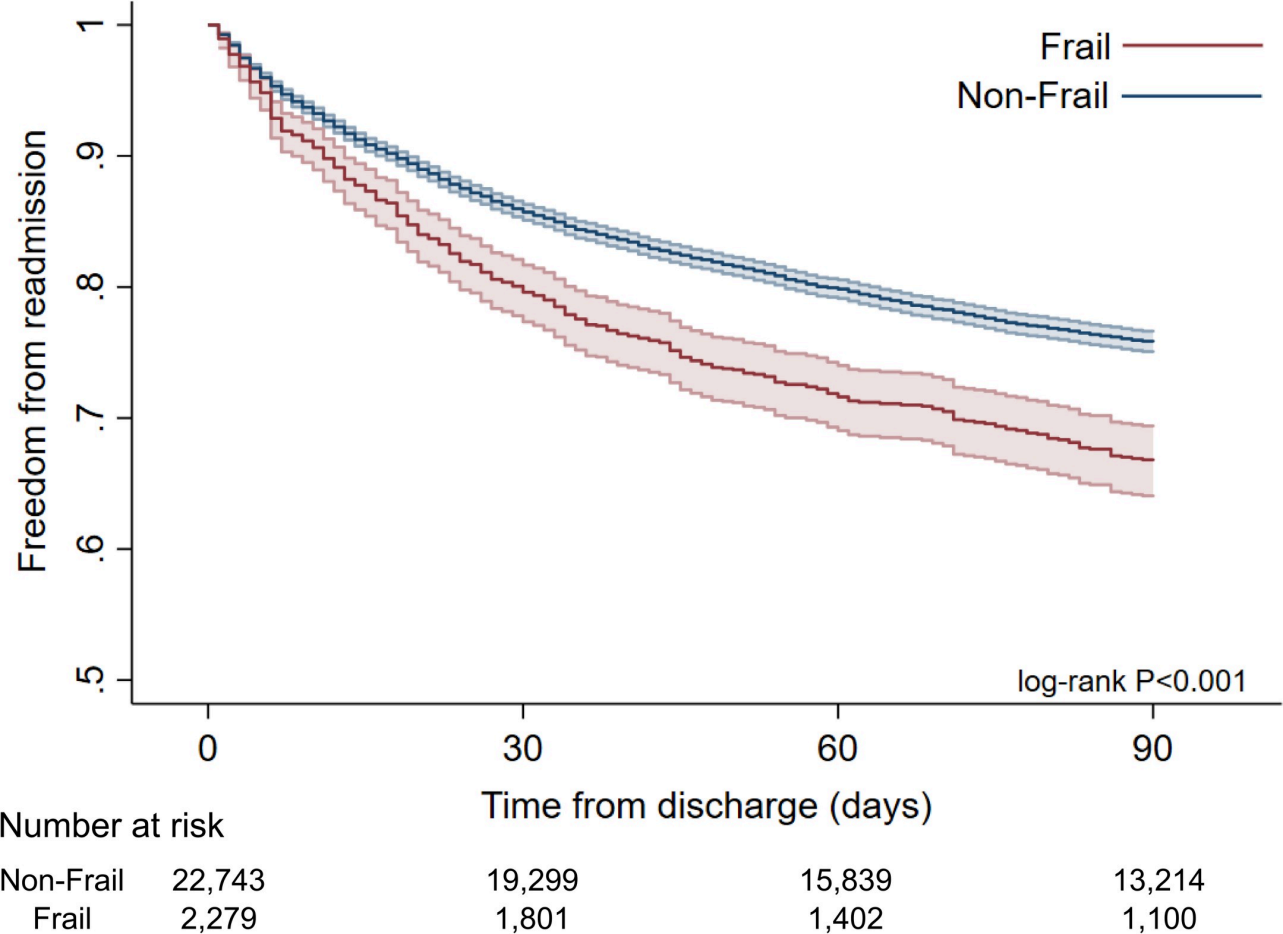
Kaplan Meier survival estimates for patients undergoing TMVR from 2016–2018 by frail and non-frail cohorts.

Unadjusted outcomes for the two groups are presented in Table 2. Mean observed mortality rate for the entire cohort was 2.2%. Compared to their counterparts, Frail patients had higher unadjusted in-hospital mortality (6.0% vs 1.7%, P<0.001). In addition, Frail patients had higher unadjusted rates of cardiovascular, respiratory, renal and infectious complications (Table 2). Median costs of index hospitalization were significantly greater in the Frail relative to Non-Frail group ($55,200, IQR 38,300–85,400 vs $41,400, IQR 31,000–55,500, P<0.001). Postoperative length of stay was significantly greater in the Frail compared to the Non-Frail (6.3±8.8 days vs 2.7±3.8 days, P<0.001). Among those surviving the index admission, Frail patients had a higher proportion of nonhome destinations (41.1% vs 9.65%, P<0.001). Finally, Frail had a greater proportion of readmissions within 90 days (23.3% vs 17.1%, P<0.001) (Fig 1). As demonstrated in Table 3, the most common reasons for readmission were cardiovascular, fluid/electrolyte, and infectious reasons, with a similar distribution in Frail and Non-Frail groups.

**Table 2:** Unadjusted outcomes of patients undergoing TMVR from 2016–2018 by frail and non-frail cohorts.

	*Frail* (n = 2,179)	*Non-Frail* (n = 16,612)	*P-value*
In-Hospital Mortality (%)	5.98	1.69	<0.001
Complications (%)			
Cardiac	11.1	4.94	<0.001
Pulmonary	17.7	5.02	<0.001
Infectious	7.54	1.41	<0.001
Renal	31.6	11.8	<0.001
Non-home Discharge	41.1	9.65	<0.001
Readmission at 90-days	23.3	17.1	<0.001
Postoperative length of stay (SD)	6.3 (8.8)	2.7 (3.8)	<0.001
Hospitalization Cost (IQR)	55.2 (38.3–85.4)	41.1 (31.0–55.5)	<0.001

Nonhome discharge location includes short-term hospital, skilled nursing facility, or intermediate care facility. Hospitalization costs reported in $1,000 US Dollars and length of stay reported in days.

**Table 3:** Primary readmission diagnoses for patients readmitted following TMVR from 2016–2018 by frail and non-frail cohorts.

Readmission Diagnoses (%)	*Frail* (n = 477)	*Non-Frail* (n = 2,798)	*P-Value*
Neurologic	3.0	4.0	0.42
Psychiatric	1.4	0.3	0.02
Cardiovascular	46.9	48.0	0.76
Pulmonary	7.8	6.9	0.60
Fluids, electrolytes, gastrointestinal	8.6	11.9	0.15
Genitourinary	3.6	1.9	0.11
Infectious	8.7	9.1	0.87
Hematologic	3.2	3.1	0.94
Endocrine	4.0	2.0	0.10
Musculoskeletal	4.0	3.5	0.67

Readmission diagnoses reported as percentage readmitted relative to total readmitted per study cohort.

### Impact of frailty on risk adjusted outcomes following TMVR

**Fig 2:**
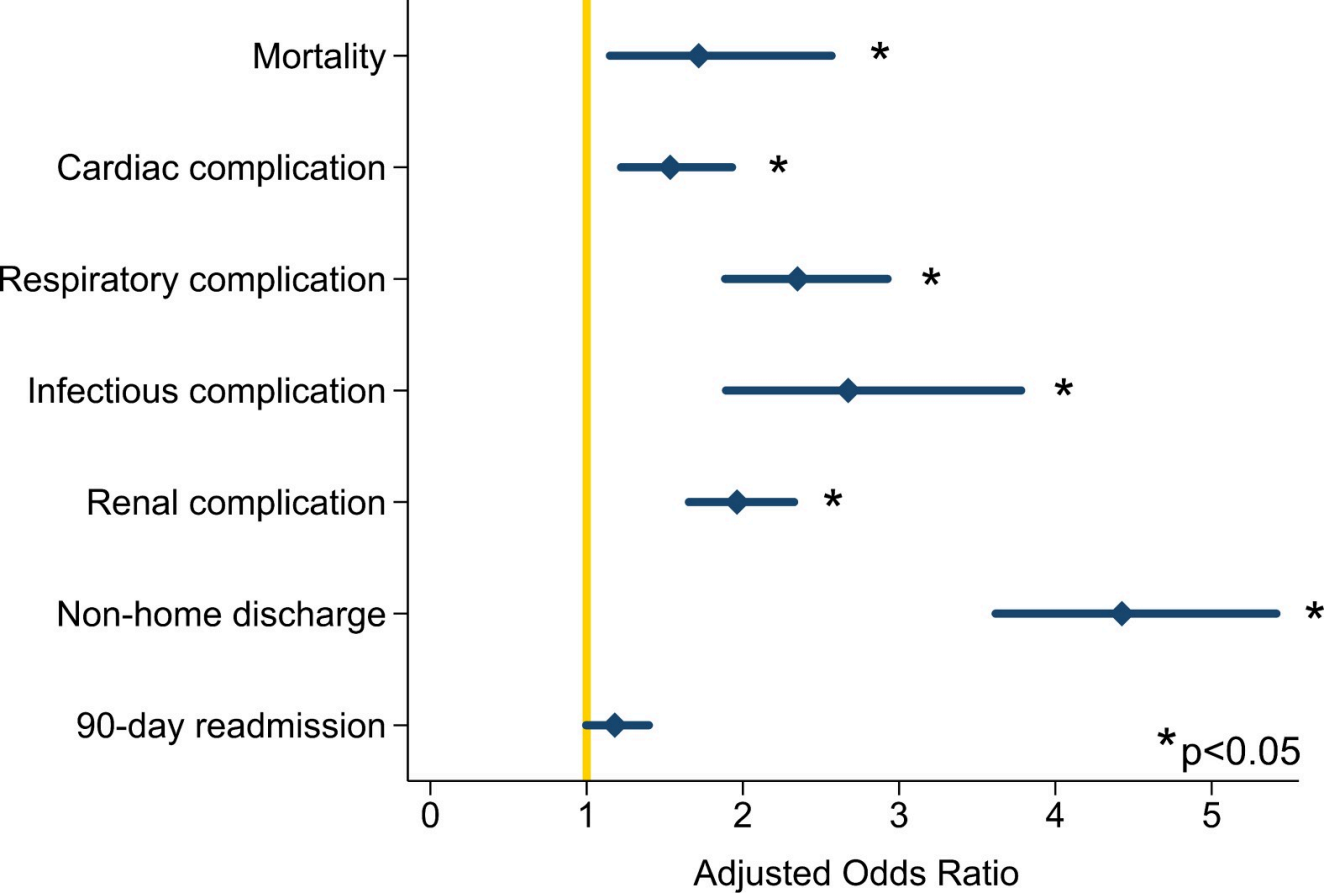
Impact of frailty on risk-adjusted outcomes. Outcomes presented as odds ratio with 95% confidence interval for *Frail* relative to *Non-Frail*. C-statistic: mortality (0.83), cardiac complication (0.72), pulmonary complication (0.78), infectious complication (0.84), renal complication (0.80), non-home discharge (0.81) and 90-day readmission (0.63). All multivariable models included adjustment for age, year, sex, chronic lung disease, diabetes, hypothyroidism, end stage renal disease, malignancy, payer status, income quartile, hospital bed size, elective admission and Elixhauser Comorbidity Index.

After adjustment for patient and hospital characteristics, frailty was associated with increased odds of in-hospital mortality (AOR 1.7, 95% CI 1.2–2.6), corresponding to an average marginal effect of 1.1% (95% CI 0.3–1.8, Fig 1). Other factors predictive of in-hospital mortality included liver disease with an average marginal effect of 3.5% (95% CI 2.3–4.6), congestive heart failure with an average marginal effect of 1.9% (95% CI 0.5–3.3), and Elixhauser Comorbidity Index ≥ 10 with an average marginal effect of 4.0% (95% CI 1.2–6.9). As demonstrated in Fig 2, frailty was associated with increased odds of all studied perioperative complications.

Relative to *Non-Frail*, *Frail* patients had a 2.7-day (95% CI 2.1–3.2) increase in adjusted postoperative length of stay. Likewise, frailty was associated with an increase of $18,300 (95% CI 14,400–22,200) in hospitalization costs. Among patients who survived to discharge, frailty was associated with increased odds (AOR 4.4, 95% CI 3.6–5.4) of nonhome discharge. Finally, adjusted odds of 90-day readmission was similar among cohorts.

## Discussion

An existing body of literature has reported frailty to be associated with worse outcomes in heart failure, open cardiac operations and transcatheter aortic valve replacement [16, 29, 30]. Although TMVR may be a viable alternative to surgical repair in high risk patients, the impact of frailty in this group has not been previously examined in detail. We used the largest publicly available database to investigate the effect of frailty, as assessed using a simple, administrative coding-based tool, on outcomes following TMVR and make several important observations. After adjusting for available patient and hospital characteristics, frailty remained a major independent predictor of mortality among patients undergoing TMVR. Similarly, frailty was associated with prolonged hospitalization and resource use as well as the need for post-discharge medical care but not readmissions. Our findings add to the existing body of literature on frailty and have important implications for patient selection and counseling. Furthermore, the present study demonstrates the ability of a coding-based frailty tool to provide additional prognostic value to existing administrative risk models.

Despite its recognized impact on postprocedural outcomes, measures of frailty have not been routinely incorporated into clinical practice. While frailty assessment tools exceed 30 in number, most are time intensive and require additional resources to administer [13, 31, 32]. Furthermore, the accuracy of such instruments has been questioned with a study demonstrating disagreement rates of 35–74% among various methods [33]. Administrative data provides a readily available alternate means to assess risk profiles including frailty. The commonly used HFRS score, derived from the National Health Services in England, provides a numeric score for frailty that has been examined in several studies [19]. While it provides acceptable discriminatory power and is associated with a differential in midterm outcomes, the HFRS has several shortcomings that may limit its utility in the preoperative setting. Firstly, dichotomization of this continuous score is arbitrary with thresholds that are likely to vary between procedure and investigators. Furthermore, the HFRS diagnostic codes include many that may be in fact postoperative complications. Thus, the prospective ability of this score to forecast procedural and surgical outcomes is uncertain. In the present study, we utilized a dichotomous frailty variable derived from a cluster of diagnostic codes as initially developed by the Johns Hopkins investigators. While the exact methodology is proprietary, we utilized codes validated in other studies to perform this analysis. This algorithm provided independent discriminatory power in our group of TMVR patients and was associated with mortality and several other endpoints of the present study. The ICD codes used in the ACG algorithm are conditions in several domains that are chronic, do not overlap with traditional surgical or procedural risk factors, and are unlikely to be related to acute hospitalization [14, 20, 21]. Whether a coding-derived frailty indicator can improve prospective determination of procedural risk warrants further investigation.

A previous single-center study of 213 patients by Metze et al. found the number of frail patients receiving TMVR was 10 times higher than rates of frail patients receiving surgical mitral valve repair, supporting the likelihood that patients not considered appropriate candidates for conventional surgical repair will be referred for percutaneous intervention [34]. While the study found frail patients to have comparable initial device success rates to non-frail patients, midterm mortality and heart failure were significantly greater in frail patients during follow-up. Poorer midterm outcomes in frail patients may be attributable to the increased risk for overall and individual postoperative complications observed in the present study. Furthermore, the association between frailty and increased LOS, hospitalization costs, and nonhome discharge observed in our study reflect the intensity of care required by frail patients beyond discharge. Previous studies have shown frailty to be associated with physical derangements such as malnutrition, weight loss, and dementia, factors that may reduce the ability to withstand perioperative stressors [11]. These factors may predispose frail patients to increased risk for complications, thereby increasing midterm mortality [31]. Interestingly, frailty was not associated with readmissions after risk-adjustment, suggesting that readmission in this patient population is driven by other factors, such as congestive heart failure, kidney dysfunction, and a greater burden of comorbidities. The present study provides the first nationwide analysis of index hospitalization for TMVR that may further forecast inferior long-term outcomes in frail patients receiving this procedure. Given the paucity of available data, further investigation of specific frailty-associated comorbidities that enhance procedural risk in the TMVR patient population is warranted.

The aforementioned study by Metze et al. identified 45.5% of patients as frail. Another recent analysis of TMVR outcomes using the Society of Thoracic Surgery Transcatheter Valve Therapy Registry by Sorajja et al. found a similarly high incidence of frailty, at 50.3% [35]. In contrast to these findings, our study identified frailty in comparatively less patients at approximately 12%. Beyond differences in sample size, this variation may be attributable to the differing methodologies used to identify frail patients. Metze et al. utilized the Fried criteria, which comprises five clinical characteristics including weakness, unintentional weight loss, exhaustion, slow gait, and low physical activity [36]. The Johns Hopkins ACG frailty indicator likewise provides a comprehensive and standardized procedure for identifying frail patients. In addition to substantiation by previous surgical studies of patient outcomes, it has been externally validated using the Vulnerable Elderly Scale and has been shown to accurately capture patients with limitations in activities of daily living [37]. Furthermore, its utilization of ICD- coding offers a uniform method of assessing frailty in the absence of granular clinical characteristics relied upon by other frailty indices, such as the Fried criteria. As frailty encompasses a broad range of clinical diagnoses, it is important that future studies of percutaneous repair adopt a consistent approach towards procedural risk in TMVR candidates. This standardization may better inform shared decision making in patients who are at particularly high general risk of deteriorating quality of life.

### Limitations

The present study had several important limitations. The administrative nature of the NRD precludes access to granular physiologic and echocardiographic parameters such as ejection fraction and intracardiac pressures as well as New York Heart Association class. Moreover, diagnoses and procedures were identified using ICD-10 codes, which are dependent on hospital coding practices and may be prone to bias. However, we selected ICD-10-CM codes that were either previously validated or clinically relevant to inform multivariable models. Additionally, we utilized robust statistical methods, such as Elastic Net for variable selection, to reduce bias within the confines of available data. Furthermore, the data in the present study was limited to in-hospital outcomes and, as such, are unable to comment on the impact of frailty on long-term outcomes. Nonetheless, the NRD is unique in providing accurate estimates for all US hospitalizations and information on resource utilization, a facet not provided by registry data.

## Conclusion

We used a large nationally representative cohort of TMVR patients and found frailty to be independently associated increased mortality, complications and resource utilization. Frailty further was associated with the need for post-discharge medical care but not readmission. Frailty derived from an administrative coding-based tool appears to serve as a powerful predictor of perioperative benchmarks, and may better inform shared decision making. With increased adoption of transcatheter therapies, incorporation of frailty into existing risk models should be strongly considered.
